# Supplementary material for: Phosphate-binding pocket on cyclin B governs CDK substrate phosphorylation and mitotic timing
Source: Nat Commun. 2025 May 8;16:4281. doi: 10.1038/s41467-025-59700-7 (PMC12062237; doi:10.1038/s41467-025-59700-7)
Supplement: Supplementary file 1 — Supplementary Information [file 41467_2025_59700_MOESM1_ESM.pdf]

Supplementary Information

Phosphate-binding pocket on cyclin B governs CDK substrate phosphorylation and mitotic timing

Henry Y. Ng, Devon H. Whelpley, Armin N. Adly, Robert A. Maxwell, and David O. Morgan

Supplementary Figures 1-9

Supplementary Tables 1, 2

Supplementary References

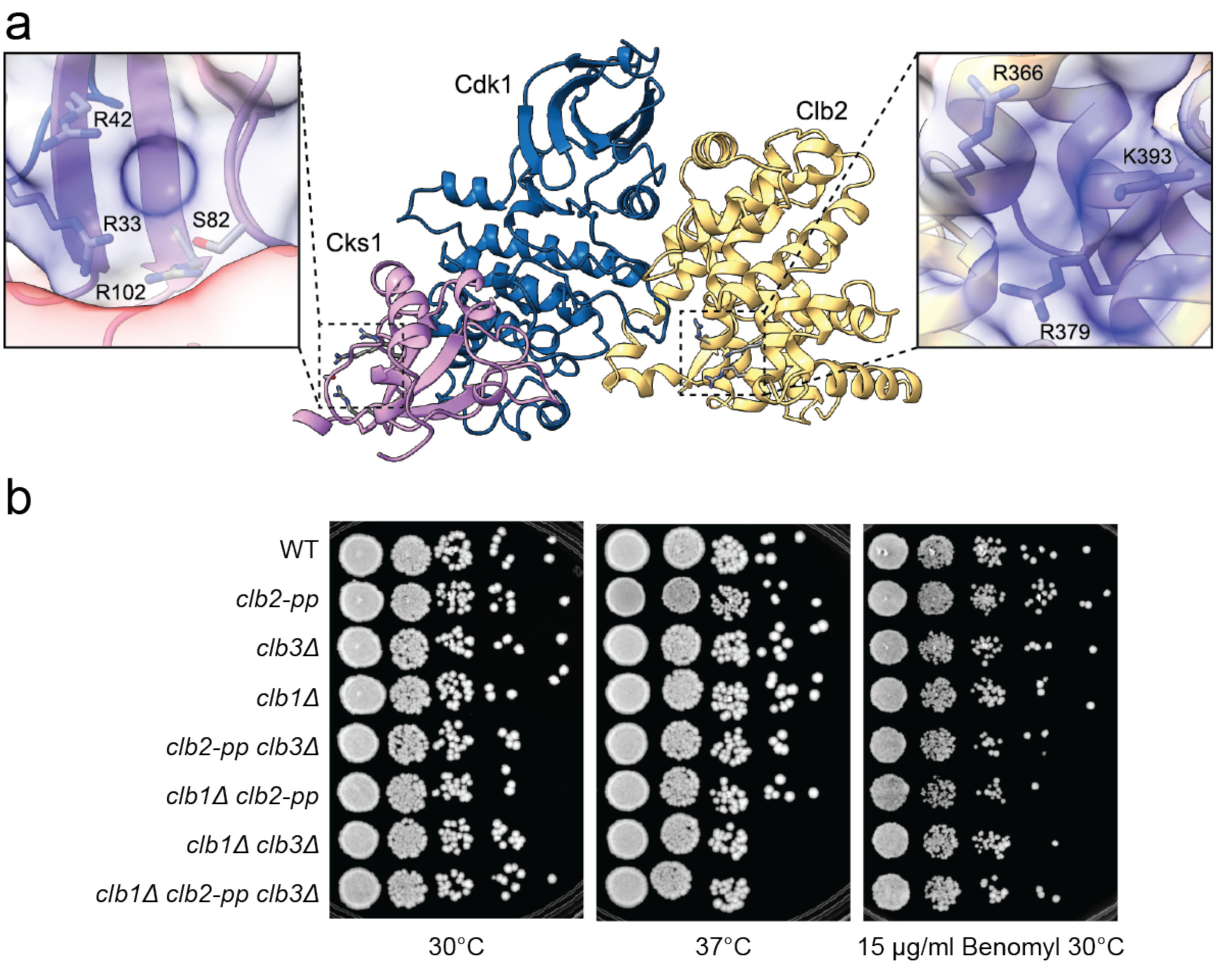

Supplementary Fig. 1: Mutation of the conserved phosphate-binding pocket in Clb2 has little effect on yeast colony growth on plates.

a, Alphafold structure model of yeast Cdk1-Clb2-Cks1 complex. Insets show electrostatic surfaces of phosphate-binding pockets in Cks1 (left) and Clb2 (right), colored blue to indicate positive charge. **b**, The indicated yeast strains (with endogenous Myc-tagged securin) were spotted on YPD plates and incubated for 48 h at the indicated temperatures. Right panel shows growth on plates containing benomyl (15 μg/ml). Each spot represents 5 μl of a yeast culture at OD_{600} = 0.1 followed by four 1:10 serial dilutions. Source data are provided as a Source Data file.

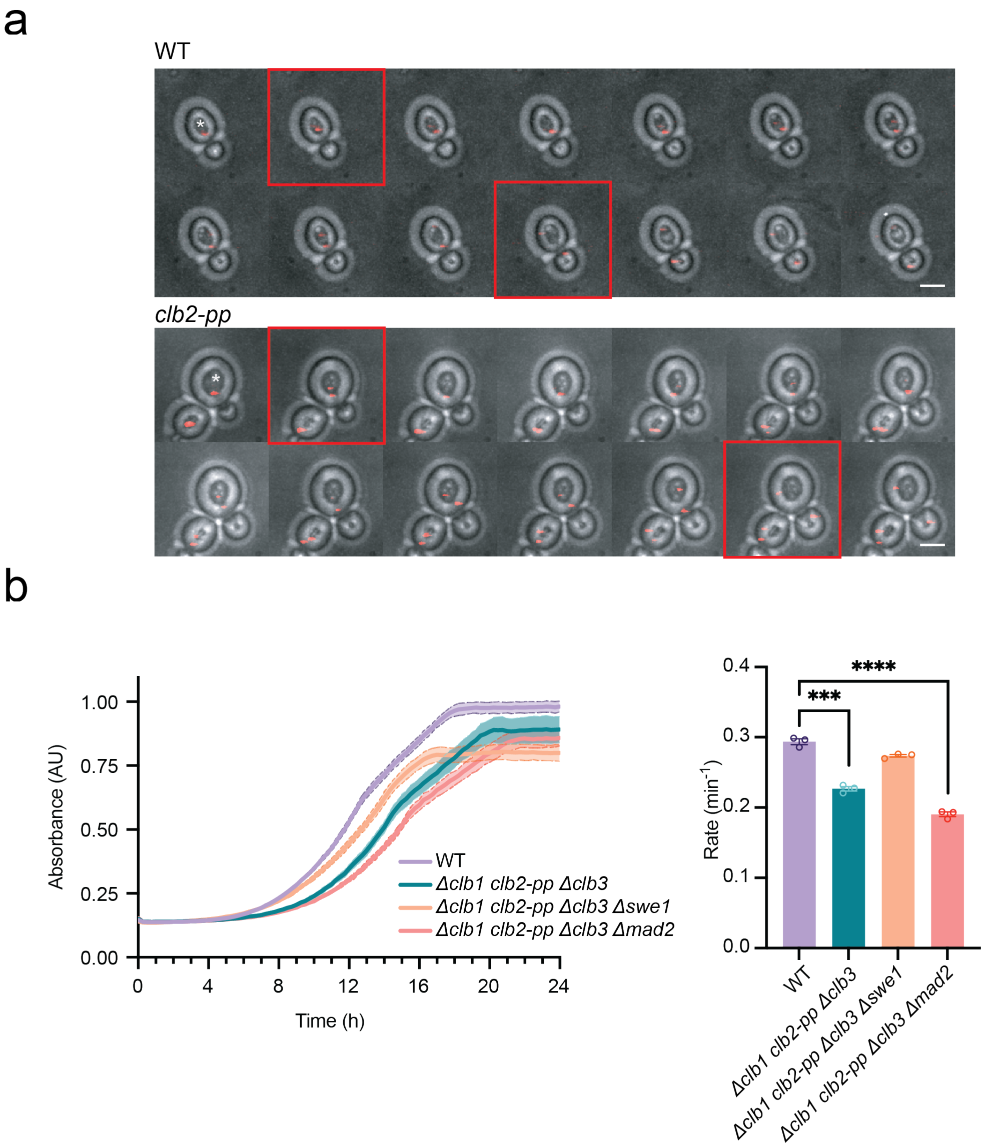

Supplementary Fig. 2: The *clb2-pp* mutation causes a mitotic delay.

a, Representative fluorescence image montages of yeast strains carrying mCherry-labeled Spc42, captured over 48 min at 2 min intervals. Images depict representative yeast cells with distinct mCherry foci (red). Frames indicated with red outlines depict (1) SPB separation and (2) initiation of spindle elongation. White asterisks mark the cells entering mitosis used for quantification. The scale bar represents 3 μm. See Fig. 2a for data from multiple cells. **b**, (left) Growth curves of indicated yeast strains, with OD_{600} recorded every 15 min. Data represent mean ± SD of three independent biological replicates. (right) Growth rates calculated by fitting OD_{600}/min to a logistic growth model. Data represent mean ± SD of three independent biological replicates. Statistical significance was determined using ANOVA (***$p<0.001$; ****$p<0.0001$). Source data are provided as a Source Data file.

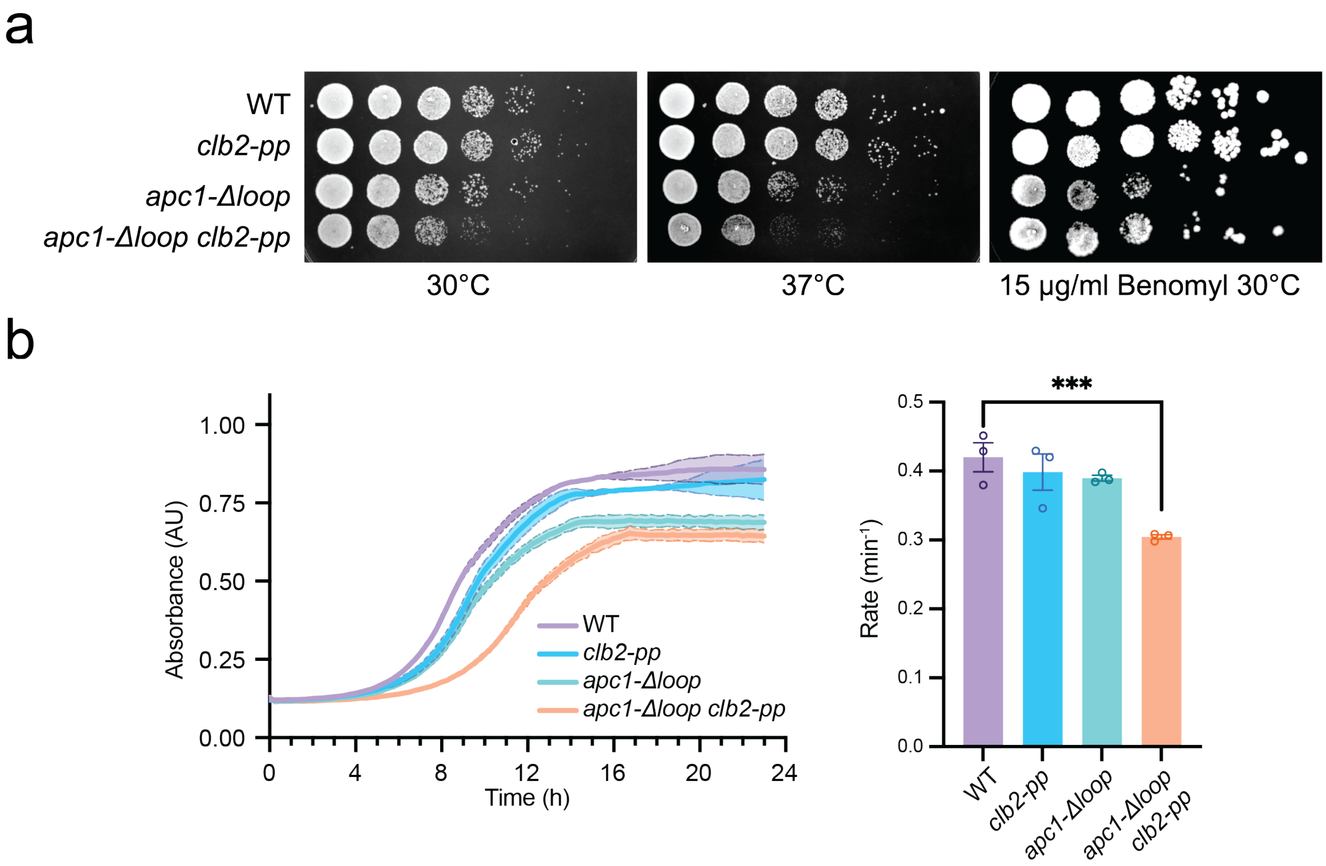

Supplementary Fig. 3: The *clb2-pp* defect is not rescued by deletion of the putative regulatory loop in Apc1.

a, The indicated yeast strains were spotted on YPD plates and incubated for 48 h at the indicated temperatures. Right panel shows growth on plates containing benomyl (15 µg/ml). Each spot represents 5 µl of a yeast culture at OD_{600}=0.1 followed by four 1:10 serial dilutions. **b**, (left) Growth curves of indicated yeast strains, with OD_{600} recorded every 15 min. Data represent mean ± SD of three independent biological replicates. (right) Growth rates calculated by fitting OD_{600}/min to a logistic growth model. Data represent mean ± SD of three independent biological replicates. Statistical significance was determined using ANOVA (***p<0.001). Source data are provided as a Source Data file.

Cdc16

```
1    MKFCLYCCHC YIVICGKATH YYKSSKATSN LKSSNRVLMR NPMSPSEQHS QHNSTLAASP FVSNVSAART QQSLPTDAQN
81   DRLQQPWNRT NTATSPYQSL ANSPLIQKLQ ANIMTPHQPS ANSNSNSNSI TGNVVNDNNL LASMSKNSMF GSTIPSTLRK
161  VSLQREYKDS VDGVVRDEDN DEDVHNNGDA AANANNDRES KLGHNGPLTT TTLTTTTTAT QLDVSELSAI ERLRLWRFDA
241  LMQHMYRTAE YIADKVYNIS NDPDDAFWLG QVYYNNNQYV RAVELITRNN LDGVNILCRY LLGLSFVKLQ RFDDALDVIG
321  EYNPFSEDPS TTAANTMSNN GNNSNTSQPV TDGGIKMESS LCFLRGKIYF AQNNFNKARD AFREAILVDI KNFEAFEMLL
401  SKNLLTPQEE WDLFDSLDFK EFGEDKEIMK NLYKINLSKY INTEDITKSN EILAKDYKLA DNVDVVRSKV DICYTQCKFN
481  ECLELCETVL ENDEFNTNIL PAYIGCLYEL SNKNKLFLLS HRLAETFPKS AITWFSVATY YMSLDRISEA QKYYSKSSIL
561  DPSFAAAWLG FAHTYALEGE QDQALTAYST ASRFFPGMHL PKLFLGMQFM AMNSLNLAES YFVLAYDICP NDPLVLNEMG
641  VMYFKKNEFV KAKKYLKKAL EVVKDLDPSS RTTISIQLNL GHTYRKLNEN EIAIKCFRCV LEKNDKNSEI HCSLGYLYLK
721  TKKLQKAIDH LHKSLYLKPN NSSATALLKN ALELNVTLSL DASHPLIDKS NLMSQASKDK ASLNKKRSSL TYDPVNMAKR
801  LRTQKEIFDQ NNKALRKGGH DSKTGSNNAD DDFDADMELE *
```

Cdc27

```
1    MAVNPELAPF TLSRGIPSFD DQALSTIIQL QDCIQQAIQQ LNYSTAEFLA ELLYAECSIL DKSSVYWSDA VYLYALSLFL
81   NKSYHTAFQI SKEFKEYHLG IAYIFGRCAL QLSQGVNEAI LTLLSIINVF SSNSSNTRIN MVLNSNLVHI PDLATLNCLL
161  GNLYMKLDHS KEGAFYHSEA LAINPYLWES YEAICKMRAT VDLKRVFFDI AGKKSNSHNN NAASSFPSTS LSHFEPRSQP
241  SLYSKTNKNG NNNINNNVNT LFQSSNSPPS TSASSFSSIQ HFSRSQQQQA NTSIRTCQNK NTQTPKNPAI NSKTSSALPN
321  NISMNLVSPS SKQPTISSLA KVYNRNKLLT TPPSKLLNND RNHQNNNNNN NNNNNNNNNN NNNNNNNNII NKTTFKTPRN
401  LYSSTGRLTT SKKNPRSLII SNSILTSDYS ITLPEIMYNF ALILRSSSQY NSFKAIRLFE SQIPSHIKDT MPWCLVQLGK
481  LHFEIINYDM SLKYFNRLKD LQPARVKDME IFSTLLWHLH DKVKSSNLAN GLMDTMPNKP ETWCCIGNLL SLQKDHDAAI
561  KAFEKATQLD PNFAYAYTLQ GHEHSSNDSS DSAKTCYRKA LACDPQHYNA YYGLGTSAMK LGQYEEALLY FEKARSINPV
641  NVVLICCCGG SLEKLGYKEK ALQYYELACH LQPTSSLSKY KMGQLLYSMT RYNVALQTFE ELVKLVPDDA TAHYLLGQTY
721  RIVGRKKDAI KELTVAMNLD PKGNQVIIDE LQKCHMQE*
```

Bud6

```
1    MKMAVDDPTY GTPKIKRTAS SSSSIETTVT KLLMSTKHLL QVLTQWSKGT TSGRLVSDAY VQLGNDFKVV SKFFMHAKVD
81   MSDVGDVPMA LRRVLEVTLR EPPSDETLNK HLPKIREIIV TLLDKLKVKQ AILKNMQQEH RISVKSHHQQ NPSFTSNLSL
161  GSEGTREGTP LSSRKSSIVR DQRQSDSVEN SYGEKVNSTS TGTPSAQSAE ATLTKPRTNI KQNLKSNNAP NASDDDDALS
241  QLKKGTNLQR RASKRYSAYH MAKLTNQSTT EAAAAAGLMT TPSPSMLHLE ETVRKSKLYG NNNNDDDRNI NSAENKGKSI
321  DDVSKASPLA KTPLPIENVR ASPRRLSSVV TTSPDKAMNG TCPVFLRIGD KTKKCHVQLP TTKNALRLLF IERFAYSPGA
401  NSFPDIYIMD PQYGVFYELE ELNLLDIKEG FVIELKLEEN PNNTIKEFID TVKMEISNSQ NDIIRHLKEM SFGSAISGKQ
481  TEVLPQPGLE ANKHDLVGQN KKDDDKTIKD IQYELGKIKQ VHNINRSNIN ETIFNILRKV DNFKSLSFSA KNSSNRMYME
561  KSQTELGDLS DTLLSKVDDL QDVIEIMRKD VAERRSQPAK KKLETVSKDL ENAQADVLKL QEFIDTEKPH WKKTWEAELD
641  KVCEEQQFLT LQEELILDLK EDLGKALETF DLIKLCCEEQ EKNPSRSKSN PILPIMRPGT FNQVREQVMV AVQSLNPDHD
721  SRVEAIDKAE KMWEMERKLK ASNEFDDELE NFVGNSNLKK SGGFEEVERI RKQKDEANLR AYFGPGFT*
```

Spa2

```
1    MGTSSEVSLA HHRDIFHYYV SLKTFFEVTG ENRDRSNSTR AQKARAKLLK LSSSQFYELS TDVSDELQRR IGEDANQPDY
81   LLPKANFHMK RNQARQKLAN LSQTRFNDLL DDILFEIKRR GFDKDLDAPR PPLPQPMKQE VSKDSDDTAR TSTNSSSVTQ
161  VAPNVSVQPS LVIPKMASID WSSEEEEEEQ VKEKPNEPEG KQTSMDEKKE AKPALNPIVT DSDLPDSQVL ARDITSMART
241  PTTTHKNYWD VNDSPIIKVD KDIDNEKGPE QLKSPEVQRA ENNNPNSEME DKVKELTDLN SDLHLQIEDL NAKLASLTSE
321  KEKEKKEEKE EKEKEKNLKI NYTIDESFQK ELLSLNSQIG ELSIENENLK QKISEFELHQ KKNDNHNDLK ITDGFISKYS
401  SADGLIPAQY ILNANNLIIQ FTTRLSAVPI GDSTAISHQI GEELFQILSQ LSNLISQLLL SADLLQYKDQ VILLKASLSH
481  AITSIRYFSV YGPVLIPKIT VQAAVSEVCF AMCNLIDSAK IKSDSNGEST TSNEGNRQVL EYSSPTATTP MTPTFPSTSG
561  INMKKGFINP RKPASFLNDV EEEESPVKPL KITQKAINSP IIRPSSSNGV PTTSRKPSGT GLFSLMIDSS IAKNSSHKED
641  NDKYVSPIKA VTSASNSASS NISEIPKLTL PPQAKIGTVI PPSENQVPNI KIENTEEDNK RSDITNEISV KPTSSIADKL
721  KQFEQSSEKK SSPKENPIAK EEMDSKPKLS NKFITSMNDV STDDSSSDGN ENDDADDDDD FTYMALKQTM KREGSKIEKN
801  NDSKLPANIV ELDLHESPES VKIESPESIK EITSSEMSSE MPSSSLPKRL VEDVEPSEMP EKGASVESVR KKNFQEPLGN
881  VESPDMTQKV KSLGMTGKAV GPESDSRVES PGMTGQIKSL NMAGKVVGPE ADSRVESPGM KEQIKSLGMT GKITAQESIK
961  SPEAARKLAS SGEVDKIESP RMVRESESLE AVGNTIPSNM TVKMESPNLK GNTVSEPQEI RRDIASSEPI ENVDPPKVLK
1041 KIVFPKAVNR TGSPKSVEKT PSSATLKKSG LPEPNSQIVS PELAKNSPLA PIKKNVELRE TNKPHTETIT SVEPTNKDAN
1121 TSWRDADLNR TIKREEEDED FDRVNHNIQI TGAYTKTGKI DYHKIPVDRK AKSEAEVHTS EEDIDESNNV NGKRADAQIH
1201 ITERKHAFVN PTENSQVKKT SHSPFLNSKP VQYENSESNG GINNHIKIKN TGETTAHDEK HYSDDDDSSY QFVPMKHEEQ
1281 EQEQNRSEEE ESEDDDEEEE DSDFDVDTFD IENPDNTLSE LLLYLEHQTM DVISTIQSLL TSIKKPQVTK GNLRGESNAI
1361 NQVIGQMVDA TSISMEQSRN ANLKKHGDWV VQSLRDCSRR MTILCQLTGD GILAKEKSDQ DYADKNFKQR LAGIAFDVAK
1441 CTKELVKTVE EASLKDEINY LNSKLK*
```

Supplementary Fig. 4: Amino acid sequences of the CDK substrates analyzed in this study. CDK consensus sequences (S/T*-P and S/T*-PxK/R) are highlighted in yellow. Highlighted in green is the disordered fragment used for kinase reactions *in vitro*. Amino acids colored in red were identified as phosphorylation sites in our mass spectrometry analysis (Supplementary Data 2, Fig. 6).

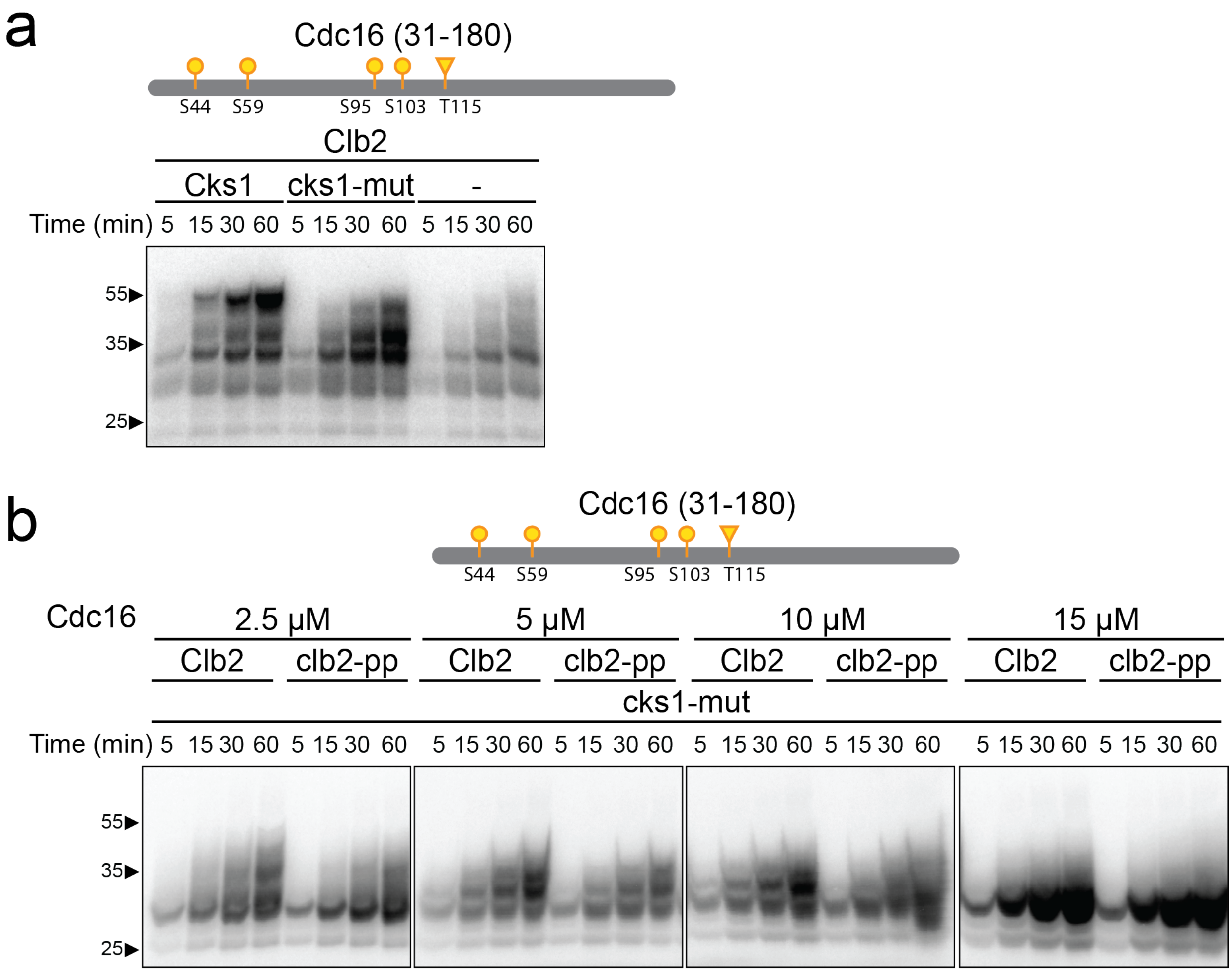

Supplementary Fig. 5: Multi-site phosphorylation of CDK substrates *in vitro* is dependent on the presence of Cks1 and substrate concentration.

a, 2.5 µM purified Cdc16 fragment (aa 31-180) was incubated with 150 nM wild-type or *clb2-pp* Clb2-Cdk1 plus wild-type, mutant or no Cks1 and radiolabeled ATP. **b**, The indicated concentrations of purified Cdc16 fragment (aa 31-180) were incubated with 150 nM wild-type or *clb2-pp* Clb2-Cdk1 plus mutant Cks1 and radiolabeled ATP. Diagrams at top indicate suboptimal (S/T*-P; yellow) CDK consensus sites (S: circle; T: triangle) in the tested fragment (see Supplementary Fig. 4 for complete sequences). Source data are provided as a Source Data file.

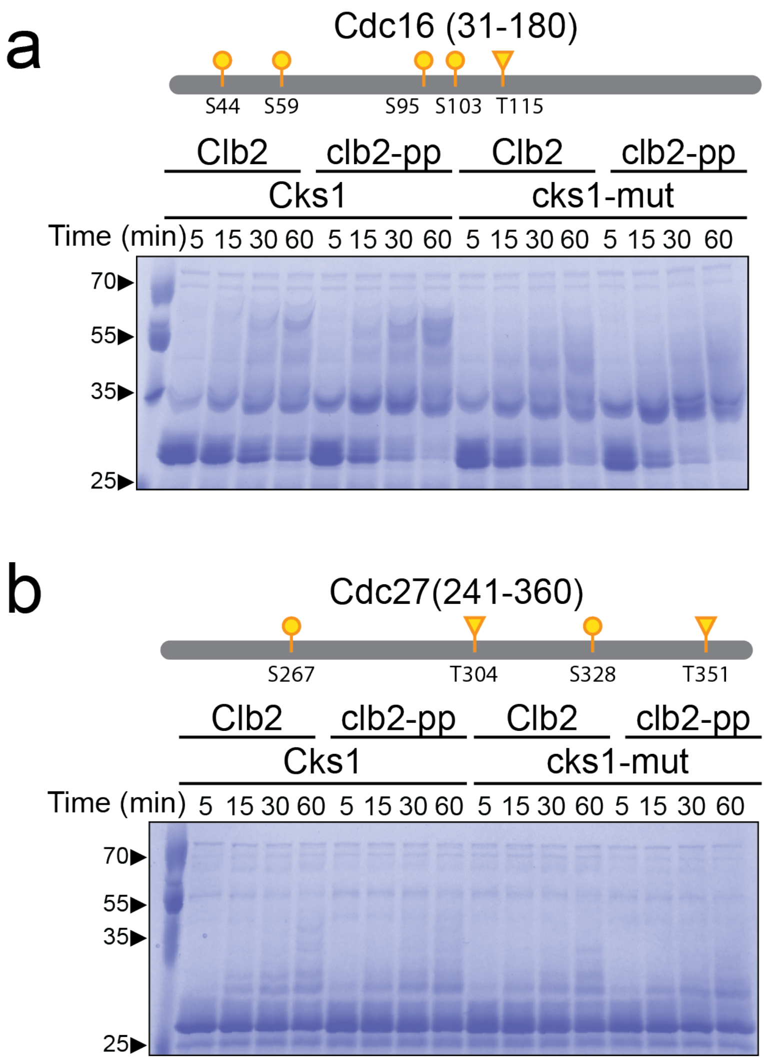

Supplementary Fig. 6: Phospho-pocket mutations reduce phosphorylation of APC/C subunits *in vitro*.

Phos-tag polyacrylamide gels for the kinase assays in Fig. 3b and c were stained with Coomassie Brilliant Blue to demonstrate extensive PP-dependent multisite phosphorylation of some substrates. Source data are provided as a Source Data file.

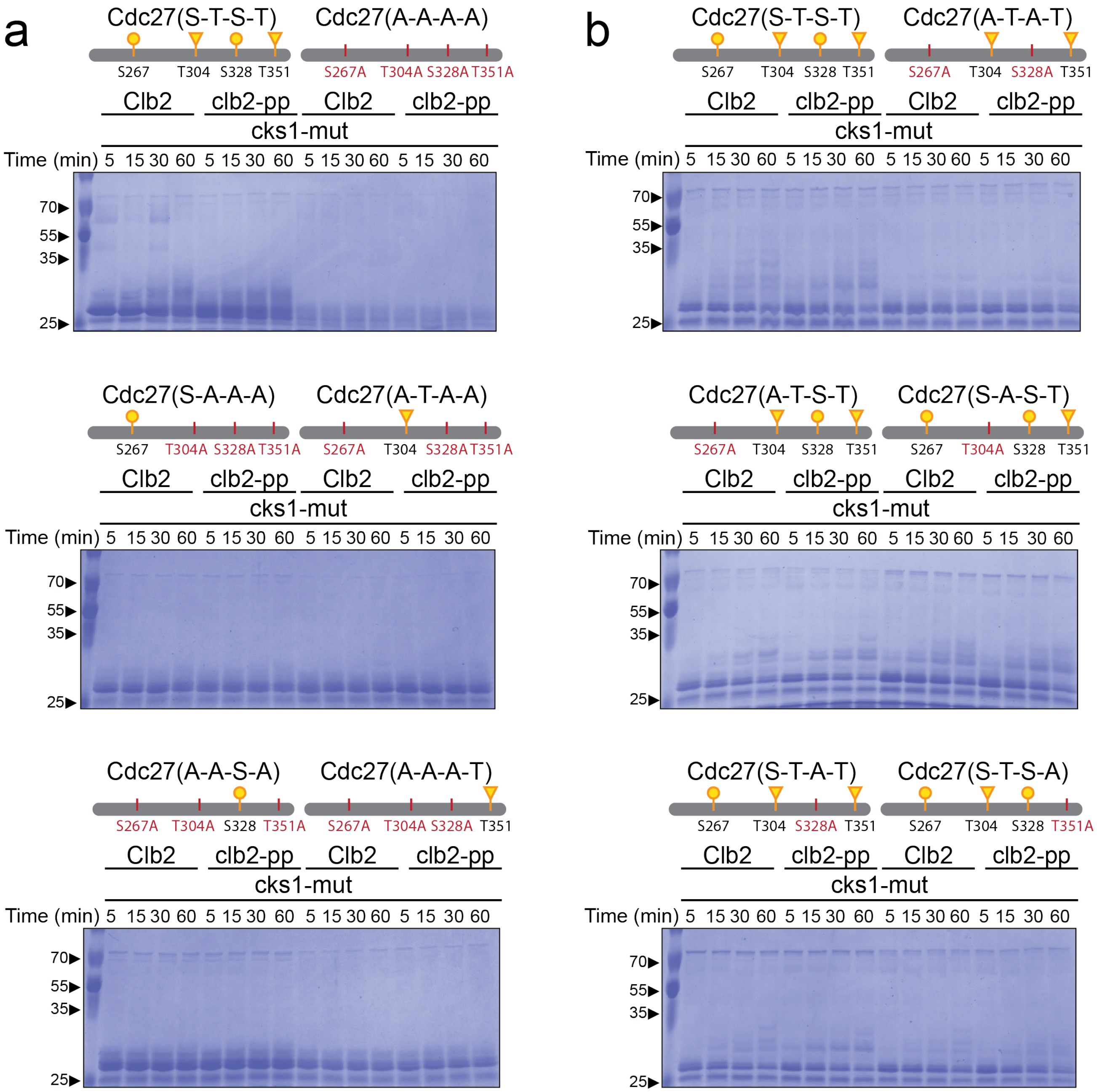

Supplementary Fig. 7: Phospho-pocket mutations reduce phosphorylation of CDK consensus sites on Cdc27.

Phos-tag polyacrylamide gels for the kinase assays in Fig. 4 were stained with Coomassie Brilliant Blue to demonstrate extensive PP-dependent multisite phosphorylation of some substrates. Source data are provided as a Source Data file.

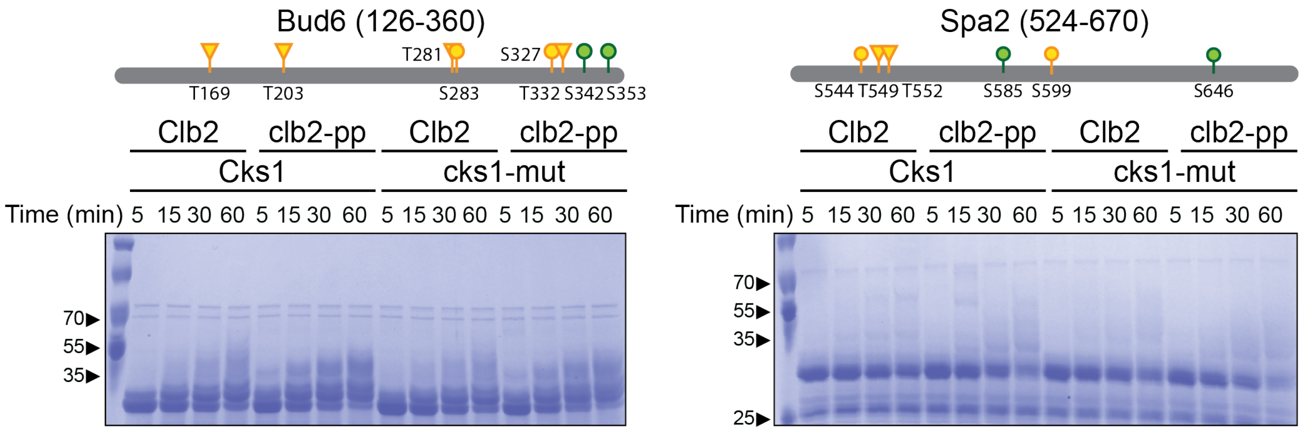

Supplementary Fig. 8: Phospho-pocket mutations reduce phosphorylation of polarisome subunits

Phos-tag polyacrylamide gels for the kinase assays in Fig. 5 were stained with Coomassie Brilliant Blue to demonstrate extensive PP-dependent multisite phosphorylation of some substrates. Source data are provided as a Source Data file.

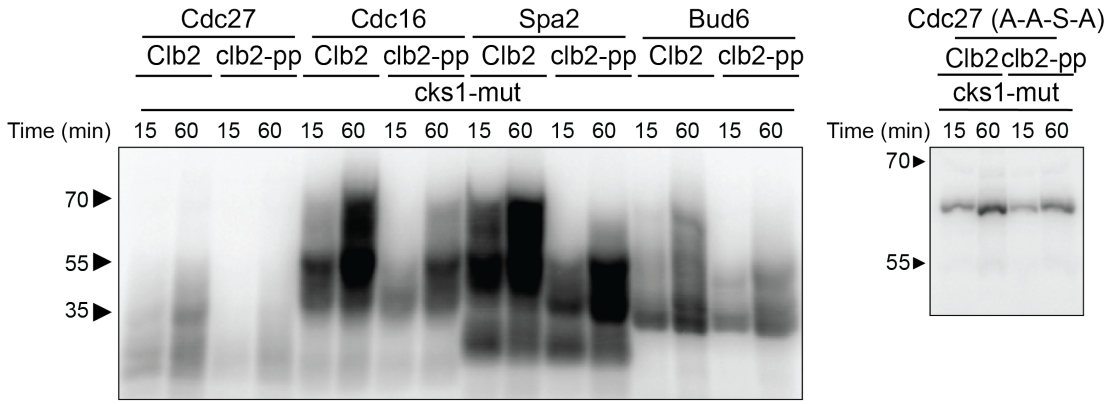

Supplementary Fig. 9: Kinase reaction products for samples analyzed by mass spectrometry.

Radiolabeled kinase reactions with the five indicated protein fragments were prepared in parallel to the unlabeled samples used for mass spectrometry analysis. Kinase concentrations were 200 nM (+ 200 nM mutant Cks1); substrate concentrations were 10 μM. Reaction products were analyzed by Phos-tag SDS-PAGE (7.5% for gel on left; 12.5% polyacrylamide for gel on right). Source data are provided as a Source Data file.

Supplementary Table 1: Yeast Strains used in this work.

Name	Genotype	Source
DOM123	*W303, MATa, bar1-HisG*	ATCC
JBA53	*trp1::pGAL-NDD1-9xMYC-TRP1*	[1]
HN40	*clb2Δ::HYGR, trp1::pGAL-NDD1-9xMYC-TRP1*	This Study
JBA71	*clb2::CLB2-R336A,R379A,K383A-HYGR, trp1::pGAL-NDD1-9xMYC-TRP1*	[1]
HN01	*clb3Δ::pAgTEF-KANMX-tAgTEF, trp1::pGAL-NDD1-9xMYC-TRP1*	This Study
HN02	*clb1Δ::pAGTEF-NATNT-tADH1, trp1::pGAL-NDD1-9xMYC-TRP1*	This Study
HN03	*clb3Δ::pAgTEF-KANMX-tAgTEF, clb2::CLB2-R336A,R379A,K383A-HYGR, trp1::pGAL-NDD1-9xMYC-TRP1*	This Study
HN04	*clb1Δ::pAGTEF-NATNT-tADH1, clb2::CLB2-R336A,R379A,K383A-HYGR, trp1::pGAL-NDD1-9xMYC-TRP1*	This Study
HN05	*clb3Δ::pAgTEF-KANMX-tAgTEF, clb1Δ::pAGTEF-NATNT-tADH1, trp1::pGAL-NDD1-9xMYC-TRP1*	This Study
HN06	*clb3Δ::pAgTEF-KANMX-tAgTEF, clb1Δ::pAGTEF-NATNT-tADH1, clb2::CLB2-R336A,R379A,K383A-HYGR, trp1::pGAL-NDD1-9xMYC-TRP1*	This Study
DOM1324	*SPC42-mCherry-HIS3*	[2]
HN24	*SPC42-mCherry-HIS3, clb2::CLB2-R336A,R379A,K383A-HYGR*	This Study
DOM1228	*pds1::PDS1-myc13-TRP1*	[3]
HN10	*pds1::PDS1-myc13-TRP1, clb2::CLB2-R336A,R379A,K383A-HYGR*	This Study
HN18	*pds1::PDS1-myc13-TRP1, clb3Δ::pAgTEF-KANMX-tAgTEF*	This Study
HN19	*pds1::PDS1-myc13-TRP1, clb1Δ::pAgTEF-NATNT-tADH1*	This Study
HN13	*pds1::PDS1-myc13-TRP1, clb3Δ::pAgTEF-KANMX-tAgTEF, clb2::CLB2-R336A,R379A,K383A-HYGR*	This Study
HN16	*pds1::PDS1-myc13-TRP1, clb1Δ::pAgTEF-NATNT-tADH1, clb2::CLB2-R336A,R379A,K383A-HYGR*	This Study
HN22	*pds1::PDS1-myc13-TRP1, clb3Δ::pAgTEF-KANMX-tAgTEF, clb1Δ::pAGTEF-NATNT-tADH1*	This Study
HN21	*pds1::PDS1-myc13-TRP1, clb3Δ::pAgTEF-KANMX-tAgTEF, clb1Δ::pAgTEF-NATNT-tADH1, clb2::CLB2-R336A,R379A,K383A-HYGR*	This Study
HN43	*pds1::PDS1-myc13-TRP1, swe1Δ::pAgTEF-NATNT-tADH1*	This Study
HN44	*pds1::PDS1-myc13-TRP1, swe1Δ::pAgTEF-NATNT-tADH1, clb2::CLB2-R336A,R379A,K383A-HYGR*	This Study
DW03	*pds1::PDS1-myc13-TRP1, swe1Δ::pAgTEF-NATNT-tADH1, clb3Δ::pAgTEF-KANMX-tAgTEF, clb1Δ::pAgTEF-NATNT-tADH1*	This Study
DW04	*pds1::PDS1-myc13-TRP1, swe1Δ::pAgTEF-NATNT-tADH1, clb3Δ::pAgTEF-KANMX-tAgTEF, clb1Δ::pAgTEF-NATNT-tADH1, clb2::CLB2-R336A,R379A,K383A-HYGR*	This Study
DOM1231	*pds1::PDS1-MYC-TRP1, mad2Δ::KAN*	[3]
HN39	*pds1::PDS1-MYC-TRP1, mad2Δ::KAN, clb2::CLB2-R336A,R379A,K383A-HYGR*	This Study
DW01	*pds1::PDS1-MYC-TRP1, mad2Δ::KAN, clb3Δ::pAgTEF-KANMX-tAgTEF, clb1Δ::pAgTEF-NATNT-tADH1*	This Study
DW02	*pds1::PDS1-MYC-TRP1, mad2Δ::KAN, clb3Δ::pAgTEF-KANMX-tAgTEF, clb1Δ::pAgTEF-NATNT-tADH1, clb2::CLB2-R336A,R379A,K383A-HYGR*	This Study
HN41	*pds1::PDS1-myc13-TRP1, apc1::APC1Δ225-365-NATNT*	This Study
HN42	*pds1::PDS1-myc13-TRP1, apc1::APC1Δ225-365-NATNT, clb2::CLB2-R336A,R379A,K383A-HYGR*	This Study
DOM900	*cdc16::CDC16-TAP-HIS3*	[4]

Supplementary Table 2: Plasmids used in this work.

Name	Description	Source
pHN56	pET28a-6xHis-SUMO-Clb2(187-491)	This Study
pHN57	pET28a-6xHis-SUMO-Clb2(187-491, R336A, R379A, K383A)	This Study
pHN58	pLIB-GST-TEV-Cdc28	This Study
pEV652	pET11a-Cks1 (untagged)	[5]
pEV653	pET11a-Cks1 mutant (R33A, S82E, R102A)	[6]
pDW5	pET28a-6xHis-SUMO-Cdc16 (31-180)	This Study
pHN44	pET28a-6xHis-SUMO-Cdc27 (241-360)	This Study
pDW10	pET28a-6xHis-SUMO-Cdc27 (241-360, S267A, T304A, S328A, T351A)	This Study
pHN61	pET28a-6xHis-SUMO-Cdc27 (241-360, T304A, S328A, T351A)	This Study
pHN62	pET28a-6xHis-SUMO-Cdc27 (241-360, S267A, S328A, T351A)	This Study
pHN63	pET28a-6xHis-SUMO-Cdc27 (241-360, S267A, T304A, T351A)	This Study
pHN64	pET28a-6xHis-SUMO-Cdc27 (241-360, S267A, T304A, S328A)	This Study
pAA29	pET28a-6xHis-SUMO-Cdc27 (241-360, S267A, S328A)	This Study
pAA25	pET28a-6xHis-SUMO-Cdc27 (241-360, S267A)	This Study
pAA26	pET28a-6xHis-SUMO-Cdc27 (241-360, T304A)	This Study
pAA27	pET28a-6xHis-SUMO-Cdc27 (241-360, S328A)	This Study
pAA28	pET28a-6xHis-SUMO-Cdc27 (241-360, S351A)	This Study
pHN47	pET28a-6xHis-SUMO-Bud6 (126-360)	This Study
pHN46	pET28a-6xHis-SUMO-Spa2 (524-670)	This Study

Supplementary References

1. Asfaha, J. B. *et al.* Multisite phosphorylation by Cdk1 initiates delayed negative feedback to control mitotic transcription. *Curr Biol* **32**, 256-263.e4 (2022).
2. Lu, D. *et al.* Multiple mechanisms determine the order of APC/C substrate degradation in mitosis. *J Cell Biol* **207**, 23–39 (2014).
3. Foster SA, Morgan DO. The APC/C subunit Mnd2/Apc15 promotes Cdc20 autoubiquitination and spindle assembly checkpoint inactivation. *Mol Cell* **47**, 921-932 (2012).
4. Carroll, C. W. & Morgan, D. O. The Doc1 subunit is a processivity factor for the anaphase-promoting complex. *Nat Cell Biol* **4**, 880–887 (2002).
5. Reynard, G. J., Reynolds, W., Verma, R. & Deshaies, R. J. Cks1 is required for G(1) cyclin-cyclin-dependent kinase activity in budding yeast. *Mol Cell Biol* **20**, 5858–5864 (2000).
6. Kõivomagi, M. *et al.* Cascades of multisite phosphorylation control Sic1 destruction at the onset of S phase. *Nature* **480**, 128–131 (2011).
