# Supplementary material for: Phosphate-binding pocket on cyclin B governs CDK substrate phosphorylation and mitotic timing
Source: Nat Commun. 2025 May 8;16:4281. doi: 10.1038/s41467-025-59700-7 (PMC12062237; doi:10.1038/s41467-025-59700-7)
Supplement: Supplementary file 2 — Description of Additional Supplementary Information [file 41467_2025_59700_MOESM2_ESM.pdf]

**Description of Additional Supplementary Files**

File Name: Supplementary Data 1
Description: *Mass spectrometry of phosphorylation sites in CDK substrates.* List of all substrate phosphopeptides identified in kinase reactions with wildtype or PP mutant kinase and each of five substrates. Full description on first tab.

File Name: Supplementary Data 2
Description: *Quantification of phosphorylation sites in CDK substrates.* High confidence phosphorylation site selection and quantification, for comparison of phosphorylation by wild-type and mutant PP kinase. Full description on first tab. Results summarized in Fig. 6.
